# Glycine, the smallest amino acid, confers neuroprotection against d-galactose-induced neurodegeneration and memory impairment by regulating c-Jun N-terminal kinase in the mouse brain

**DOI:** 10.1186/s12974-020-01989-w

**Published:** 2020-10-15

**Authors:** Rahat Ullah, Myeung Hoon Jo, Muhammad Riaz, Sayed Ibrar Alam, Kamran Saeed, Waqar Ali, Inayat Ur Rehman, Muhammad Ikram, Myeong Ok Kim

**Affiliations:** 1 Division of Life Sciences and Applied Life Science (BK 21plus), College of Natural Science, Gyeongsang National University, Jinju, 52828 Republic of Korea; 2 Department of Biochemistry, Abdul Wali Khan University Mardan, Mardan, Khyber Pakhtunkhwa 23200 Pakistan

**Keywords:** Glycine, d-galactose, Aging, Oxidative stress, c-Jun N-terminal kinase (JNK), Neuroapoptosis, Neuroinflammation, Neuroprotection

## Abstract

**Background:**

Glycine is the smallest nonessential amino acid and has previously unrecognized neurotherapeutic effects. In this study, we examined the mechanism underlying the neuroprotective effect of glycine (Gly) against neuroapoptosis, neuroinflammation, synaptic dysfunction, and memory impairment resulting from d-galactose-induced elevation of reactive oxygen species (ROS) during the onset of neurodegeneration in the brains of C57BL/6N mice.

**Methods:**

After in vivo administration of d-galactose (d-gal; 100 mg/kg/day; intraperitoneally (i/p); for 60 days) alone or in combination with glycine (1 g/kg/day in saline solution; subcutaneously; for 60 days), all of the mice were sacrificed for further biochemical (ROS/lipid peroxidation (LPO) assay, Western blotting, and immunohistochemistry) after behavioral analyses. An in vitro study, in which mouse hippocampal neuronal HT22 cells were treated with or without a JNK-specific inhibitor (SP600125), and molecular docking analysis were used to confirm the underlying molecular mechanism and explore the related signaling pathway prior to molecular and histological analyses.

**Results:**

Our findings indicated that glycine (an amino acid) inhibited d-gal-induced oxidative stress and significantly upregulated the expression and immunoreactivity of antioxidant proteins (Nrf2 and HO-1) that had been suppressed in the mouse brain. Both the in vitro and in vivo results indicated that d-gal induced oxidative stress-mediated neurodegeneration primarily by upregulating phospho-c-Jun N-terminal kinase (p-JNK) levels. However, d-gal + Gly cotreatment reversed the neurotoxic effects of d-gal by downregulating p-JNK levels, which had been elevated by d-gal. We also found that Gly reversed d-gal-induced neuroapoptosis by significantly reducing the protein expression levels of proapoptotic markers (Bax, cytochrome c, cleaved caspase-3, and cleaved PARP-1) and increasing the protein expression level of the antiapoptotic protein Bcl-2. Both the molecular docking approach and the in vitro study (in which the neuronal HT22 cells were treated with or without a p-JNK-specific inhibitor (SP600125)) further verified our in vivo findings that Gly bound to the p-JNK protein and inhibited its function and the JNK-mediated apoptotic pathway in the mouse brain and HT22 cells. Moreover, the addition of Gly alleviated d-gal-mediated neuroinflammation by inhibiting gliosis via attenuation of astrocytosis (GFAP) and microgliosis (Iba-1) in addition to reducing the protein expression levels of various inflammatory cytokines (IL-1βeta and TNFα). Finally, the addition of Gly reversed d-gal-induced synaptic dysfunction by upregulating the expression of memory-related presynaptic protein markers (synaptophysin (SYP), syntaxin (Syn), and a postsynaptic density protein (PSD95)) and markedly improved behavioral measures of cognitive deficits in d-gal-treated mice.

**Conclusion:**

Our findings demonstrate that Gly-mediated deactivation of the JNK signaling pathway underlies the neuroprotective effect of Gly, which reverses d-gal-induced oxidative stress, apoptotic neurodegeneration, neuroinflammation, synaptic dysfunction, and memory impairment. Therefore, we suggest that Gly (an amino acid) is a safe and promising neurotherapeutic candidate that might be used for age-related neurodegenerative diseases.

## Background

Aging is a major factor in a number of chronic diseases, including cardiovascular disorder, diabetes mellitus, cancer, Parkinson’s disease (PD), and Alzheimer’s disease (AD) [1]. Previous research in the field of neuroscience has revealed that the human brain deteriorates with age. For decades, it has been known that central nervous system (CNS) disorders involve extensive neuronal death leading to brain dysfunction [2, 3]. Mitochondria are considered major intracellular sources of reactive oxygen species (ROS), which play vital roles in various age-associated neurological disorders [4]. Biomolecules generated as a result of excessive ROS and elevated oxidative stress (lipids, proteins, and DNA) disturb neuronal homeostasis, eventually leading to neuronal cell death [5].

d-galactose (d-gal) is a substance that occurs naturally in the body. Chronic systemic administration of d-gal in rats and mice is widely used to induce artificial senescence to model brain aging in animals and thereby assess the antiaging potential of pharmacological agents [1]. Accumulating evidences suggests that systemic chronic administration of d-gal accelerates cognitive and memory impairment in animals [6–9]. Normally, two different enzymes, namely galactose-1-phosphate uridyl transferase and d-galactokinase, are involved in the metabolism of d-gal in the body. However, at high concentrations, d-gal induces oxidative stress in the presence of galactose oxidase [1, 10]. Previous studies have reported that d-gal treatment upregulates oxidative stress and ROS levels, which causes activation of stress kinases, leading to neurodegeneration [5, 11]. c-Jun N-terminal kinase (JNK), also known as stress-activated kinase (JNK/SAPK), is a member of the complex superfamily of mitogen-activated protein kinases (MAPKs) and is activated in response to oxidative stress to mediate neuroapoptosis [12]. Numerous compelling lines of evidence strongly suggest that JNK functions as a proapoptotic kinase in neuronal apoptosis [11]. Studies have shown that JNK inhibition/knockdown reduces neuroapoptosis of hippocampal neurons [11, 13]. More recently, a study reported that active JNK enhances neuroinflammatory responses, synaptic loss, and cognitive deficits associated with neurodegeneration [14].

Glycine (Gly) is a dietary nonessential amino acid that is present as a metabolic intermediate in all organisms. Gly has been reported to be an antioxidant agent [15]. In addition, Gly treatment significantly reduces oxidative stress markers in the infarcted regions in the brain and causes significant normalization of the latency period of conditioned (behavioral) reflexes [16, 17]. Gly has not been reported to induce significant side effects in normal subjects (including rats and healthy volunteers) except for a mild sedative state. Another study reported that Gly exerts antistress and nootropic effects [18]. Also, glycine acts as a co-agonist for the channel opening of *N*-methyl-d-aspartate subtype of ionotropic glutamate receptors (NMDARs) [19–21] and therefore, facilitates neurotransmission [22]. Recently, Amin et al. demonstrated that Gly has a neuroprotective effect against ethanol-induced oxidative stress and neurodegeneration by stimulating the PI3K/Akt signaling pathway [15].

The aims of the present study were to investigate the mechanism underlying the neuroprotective effect of Gly against d-gal-induced neurodegeneration. Herein, our results showed that Gly cotreatment (1 g/kg/day) mitigates d-gal-induced oxidative stress, neuroapoptosis, neuroinflammation, synaptic dysfunction, memory impairment, and neurodegeneration in the brains of C57BL/6N mice. Importantly, both an in vitro study and molecular docking analysis supported our hypothesis that the neuroprotective effect of Gly might be due to JNK inhibition. This remarkable protection afforded by Gly, a neuroprotective amino acid, makes it a potentially safe, cost-effective, and easily administered agent.

## Experimental section: materials and methods

### Chemicals

d-galactose, glycine (Gly), JNK inhibitor (SP600125), and MTT (3-(4,5-dimethylthiazol-2-Yl)-2, 5-diphenyltetrazolium bromide), 2, 7-dichlorodihydrofluorescein diacetate (DCFH-DA) were purchased from Sigma Chemical Co (St. Louis, MO, USA).

### Experimental animals

Male C57BL/6N mice (8 weeks; average body weight of 25–30 g) were purchased from Samtako Bio (Osan, S. Korea) and housed under a 12-h/12-h light/dark cycle in a temperature-controlled environment (20 ± 2 °C; humidity 50 ± 10%) in the animal care center of Gyeongsang National University, South Korea. The animals were provided ad libitum access to food and water. All the experimental animals were acclimatized for 1 week (7 days) in the animal care center. The experimental procedures were approved (approval ID: 125) by the animal ethics committee (IACUC) of the Division of Applied Life Sciences, Department of Biology at Gyeongsang National University, South Korea.

### Grouping of animals and drug treatment

The experimental animals were equally divided into the following four groups (*n* = 16 mice/group).

1. Control (Cont. (C)) group: mice treated with normal saline (0.9%) (i.p) as a vehicle for 60 days.
2. d-galactose (d-gal) treatment group: mice treated with d-gal (100 mg/kg/day; i/p for 60 days).
3. d-gal + glycine (Gly) co-treatment group: mice treated with d-gal + Gly (1 g/kg/day in 0.9% normal saline solution) for 60 days.
4. Gly alone treatment group: mice treated with Gly for 60 days.

All the experimental procedures were carried out in accordance with the rules established by the local ethical committee for animals of the Department of Biology, Division of Applied Life Sciences of the Department of Biology in the Gyeongsang National University.

### Behavioral Analysis

After administration of Gly and d-gal (d-gal; 100 mg/kg/day; i/p), we performed studies including the Morris water maze and Y-maze test.

### Morris water maze test

The Morris water maze (MWM) test is a well-known and established task for evaluating memory functions; therefore, we performed the MWM as described previously [5]. The apparatus consisted of a circular water tank (diameter of 100 cm; height of 40 cm) filled with water (23 ± 1 °C) to a depth of 15.5 cm that was rendered opaque by the addition of white ink. A transparent escape platform (diameter of 4.5 cm, height of 14.5 cm) was hidden 1 cm below the surface of water in the center of one quadrant. Each mouse underwent four training trials/day for 4 consecutive days using the hidden platform. The escape latency (latency to find the submerged hidden platform) of each mouse in each trial was calculated. On day 5, we performed a probe test to evaluate memory consolidation. In the probe test, each mouse was allowed to freely swim for 60 s after the platform was removed. In the probe trial, the time spent in the target quadrant (where the platform has been located during hidden platform training), i.e., the time spent swimming in the target quadrant in an attempt to find the removed hidden platform, the time spent in the other three quadrants (left, right, and opposite), and the number of platform crossings were measured. The degree of memory consolidation was represented by the time spent in the target quadrant after learning. Visual/video tracking software (SMART, Panlab Harvard Apparatus; Bioscience Company, Holliston, MA, USA) was used to record all data.

### Y-maze test

For the Y-maze task, a Y-maze apparatus (height = 20 cm, length = 50 cm, and width = 10 cm at the bottom) was used [23]. The apparatus was constructed from black-painted wood and used for the evaluation of spatial working memory. Briefly, individual mice were placed in the middle of the maze and allowed to move freely (3- to 8-min sessions) for different intervals of time. The sequence of arm entries was digitally recorded. A spontaneous alternation was defined as successive entry into the three arms in overlapping triplet sets. The alteration percentage (%) was calculated as successive triplet sets/total number of arms entries – 2 × 100. A greater percentage (%) of spontaneous alteration behavior reflected better cognitive performance.

### Collection of brain tissue and sample preparation

Briefly, after completion of drug treatment and behavior analyses, all the experimental mice (*n* = 16) were first anesthetized and then immediately sacrificed for immunofluorescence, morphological, and biochemical studies [24]. For morphological analysis, the experimental animals (*n* = 8 mice/group) were transcardially perfused with ice-cold PBS (0.01 M) followed by neutral-buffered paraformaldehyde (NBP; 4%) before the brains were postfixed in NBP (4%; 48–72 h). Next, the post-fixed brains of all the experimental mice were washed with 1% PBS (0.01 M) after removal of NBP and transferred into 20% sucrose solution (48 h) until they sank to the tube bottom. Finally, brains were frozen in O.C.T. compound (A.O. USA) before taking coronal cortical and hippocampal sections (14 μM) using microscopic slide via CM-3050CCryostat (Leica, Germany). The sections were thaw mounted on probe-on plus charged slides (Fisher, Rockford, IL, USA). Similarly, for biochemical or Western blotting (*n* = 8 mice/group), the brains were immediately removed, cortex and hippocampus tissue were carefully dissected, frozen on dry ice before stored at − 80 °C. After homogenization in 0.2 M PBS with phosphatase inhibitor and protease inhibitor cocktail, these samples were centrifuged at 10,000 g at 4 °C for 30 min. The supernatants were collected and stored (− 80 °C).

### MTT and cell viability assays

The viability of treated mouse hippocampal neuronal HT22 cells was assessed by a colorimetric MTT (3-[4,5-dimethylthiazol-2-yl]-2,5-diphenyl tetrazolium bromide) assay according to the manufacturer’s instructions (Sigma Aldrich) to evaluate the effect of d-gal and/or Gly. The cells were cultured in 96-well plates (density = 1 × 10^4^ cells/well; in Dulbecco’s modified Eagle’s medium (DMEM; 100 μl, Gibco, Life Technologies, USA)). After 24 h, the medium of the attached cells was refreshed with the indicated concentrations of d-gal (d-gal; 10, 50, 100, or 200 mM) and Gly (Gly; 10, 20, 30, and 40 μg/μL). The control cells were cultured in DMEM (0.01%) only. The cells were then further incubated for an additional 24 h. After being cultured for 24 h, the cells were incubated with 20 μL of MTT solution (5 mg/mL) for another 4 h at 37 °C. Subsequently, we removed the medium (50 μL) containing MTT solution from each well and added DMSO (100 μL/well). Finally, the 96-well plate was gently agitated on a shaker (10–20 min), and the absorbance was measured at 570 nm to evaluate the percentage of cell survival in all treatment groups using an ApoTox (Promega) instrument. The experiments were carried out in triplicate.

### In vivo oxidative stress and lipid peroxidation assays

The reactive oxygen species (ROS) assay was performed according to a previously described procedure with some minor modifications [5]**.** This assay involved the conversion of 2′7′-dichlorodihydrofluorescein diacetate (DCFH-DA) to 2′7′-dichlorofluorescein (DCF) via oxidation. Briefly, a final concentration of 2.5 mg tissue/500 μL was obtained by diluting brain homogenates with ice-cold Lock’s buffer (1:20). Next, the reaction mixture was incubated with Lock’s buffer (1 mL, pH = 7.4), 0.2 mL of homogenate, and 10 mL of DCFH-DA (5 mM) at room temperature for 15 min, resulting in the formation of DCF (fluorescent product) from DCFH-DA. Using a spectrofluorimeter, the converted fluorescent product, DCF, was measured (excitation/emission at 484/530 nm). To eliminate the background fluorescence (conversion of DCFH-DA in the absence of homogenate), blank samples were measured in parallel. ROS levels were measured as pmol DCF formed per minute per mg of protein.

Similarly, they lipid peroxidation LPO assay was performed to evaluate oxidative stress, as previously described [23]. The oxidative degradation of lipids can be quantified by measuring the free malondialdehyde (MDA) level**.** The level of MDA (a marker of LPO) was measured in the brain tissue homogenates using a lipid peroxidation (MDA) colorimetric/fluorometric assay kit (Bio Vision, USA, Cat # K739-100) according to the manufacturer’s protocol.

### In vitro oxidative stress and lipid peroxidation assay

The in vitro ROS assay was conducted on mouse hippocampal neuronal HT22 cells as previously described [25]. Briefly, the cells were cultured in 96-well plates in DMEM (200 μL/well) supplemented with fetal bovine serum (FBS) (10%) and penicillin/streptomycin (1%). After 24 h of incubation in a humidified incubator (5% CO_2_) at a temperature of 37 °C, the cells were treated with d-gal (100 mM), and d-gal (100 mM) with different concentrations of Gly (10, 20, 30, and 40 μg/μL) for 24 h. Subsequently, after treatment for 24 h, the cells were again exposed to DCFH-DA (50 μM/well) dissolved in DMSO/PBS and then incubated at 37 °C for 30 min. The relative absorbance of the plates (ROS-positive cells) was measured with the ApoToxGloTM assay (Promega Corp., Madison, WI, USA) at 484/530 nm. The results were expressed as pmol DCF formed/min/mg of protein in the cell lysate.

The in vitro LPO assay was performed to evaluate oxidative stress in HT22 cells as previously described [26]. A commercially available kit (Bio Vision Incorporated; catalog # K739-100) was used for the LPO assay. Briefly, mouse hippocampal neuronal HT22 cells (2 × 10^4^/mL) were cultured in DMEM in 96-well plates and incubated in a humidified incubator (5% CO_2_) at 37 °C. The cells were treated for 24 h by replacing the medium with fresh DMEM containing d-gal (100 mM d-gal) or d-gal + Gly (100 mM d-gal + 20 μg/μL Gly). Finally, LPO analysis was performed as described for earlier in vivo experiments.

### Western blot and confocal microscopy analysis of in vitro cultures following drug treatment

HT-22 is an immortalized mouse hippocampal cell line (Millipore, US). HT22 cells were cultured in Dulbecco’s modified Eagle’s medium (DMEM; Gibco, Themo Fisher Scientific) supplemented with 10% FBS and 1% antibiotic/antimycotic in a humidified incubator containing 5% CO_2_ at 37 °C [27]. After the cell reached almost 70–80% confluence, they were treated with d-gal (100 mM d-gal), d-gal + Gly (100 mM d-gal + 20 μg/μL Gly), d-gal + JNK-specific inhibitor (100 mM d-gal + 20 μM SP600125), or d-gal + Gly + JNK-specific inhibitor (100 mM SP600125 + 20 μg/μL Gly + 20 μM SP600125) for 24 h.

For Western blot analysis, mouse hippocampal neuronal HT22 cells were washed, collected, and centrifuged in 0.01 M phosphate buffer saline (PBS; Amresco, Life Sciences, USA) to remove the supernatant. Then, PRO-PREP solution was used to dissolve the remaining pellets according to the manufacturer’s protocol (iNtRON Biotechnology, Burlington, NJ, USA) before sonication to obtain cell lysates. Finally, Western blot analysis was performed as described for the in vivo experiments.

Similarly, for immunofluorescence analysis, fixed mouse hippocampal neuronal HT22 cells were washed with 0.01 M phosphate buffer saline (PBS; Amresco, Life Sciences, USA). Next, proteinase K and blocking solution were used. Primary antibodies were applied, and the cells were incubated at 4 °C overnight. After washing, secondary antibodies conjugated to FITC/TRITC (Santa Cruz Biotechnology) were then added at room temperature (1–2 h). The slides were washed twice with PBS for 5–7 min. Next, for nuclear staining, DAPI (4′,6-diamidino-2-phenylindole) was used, and glass coverslips were mounted on the slides with mounting medium.

Finally, confocal laser scanning microscopy (Flu-View FV1000 Olympus, Japan) and ImageJ software were used to examine the stained slides (by capturing images) and analyze the integral optical density (IOD), respectively.

### In vivo Western blot analysis and immunofluorescence

Western blot analysis was performed as previously performed by our laboratory [28]**.** The concentration of protein was quantified (BioRad protein assay kit, BioRad Laboratories, CA, USA). Equal amounts of protein (15–30 μg) were electrophoresed on 4–12% Bolt™ Mini Gels (Novex; Life Technologies, Kiryat Shmona, Israel). To confirm equal sample loading, an anti-β-actin antibody (Santa Cruz Biotechnology, Dallas, TX, USA) was used as a standard for comparison. After the proteins were transferred to polyvinylidene difluoride (PVDF) membranes, the membranes were blocked with skim milk 5% (w/v) or BSA to reduce nonspecific binding and then incubated with primary antibodies overnight at 4 °C. After incubation with the primary antibodies (1:1000) in Tris-buffered saline with Tween (TBST), the membranes were washed with 1× TBST (three times) and exposed to secondary antibodies (1–2 h). After reaction with a horseradish peroxidase-conjugated secondary antibody, ECL detection reagent (Amersham Pharmacia Biotech, Uppsala, Sweden) was used for visualization of proteins on X-ray films according to the manufacturer’s instructions. The developed X-ray films were scanned. ImageJ and GraphPad Prism 6 software were used for densitometric analysis of the bands and to generate histograms. Density values are expressed in arbitrary units (A.U.) relative to the untreated control.

Immunofluorescence staining was performed as previously described with some modifications [5]. For immunofluorescence staining, slides containing brain sections were washed in 0.01 M PBS (0.01 M) for 10 min. Then, the slides were incubated for 1 h in blocking solution (2% normal serum based on the corresponding antibody and 0.3% Triton X-100 in PBS). After blocking, the slides were incubated with primary antibodies (1:100 ratio in 1% PBS, i.e., 0.01 M) overnight at 4 °C followed by tetramethylrhodamine isothiocyanate (TRITC)/fluorescein isothiocyanate (FITC)-conjugated secondary antibodies (1:50 dilution in 1% 0.01 M PBS) for 2 h. DAPI (4′,6-diamidino-2-phenylindole dihydrochloride) was used to stain the nuclei (7–10 min). Finally, the slides containing brain sections were covered with glass coverslips by using mounting media. The staining patterns were examined using a confocal laser scanning microscope (Flouview FV 1000, Olympus, Japan).

### Antibodies

All antibodies used in the present study are summarized in Table 1.

**Table 1:** List of primary antibodies and their detailed information used for Western blot and immunofluorescence

Antibody	Host	Catalog	Application	Dilution	Manufacturer
Anti-**Β**-Actin antibody	Mouse	sc-47778	WB	1:1000	Santa Cruz Biotechnology (Dallas, TX, USA)
Anti-p-JNK antibody	Mouse	sc-6254	WB& IF	1:1000/1:100	Santa Cruz Biotechnology (Dallas, TX, USA)
Anti-Bax antibody	Mouse	sc-7480	WB	1:1000	Santa Cruz Biotechnology (Dallas, TX, USA)
Anti-Bcl2 antibody	Mouse	sc-7382	WB	1:1000	Santa Cruz Biotechnology (Dallas, TX, USA)
Anti-PSD-95 antibody	Mouse	sc-71933	WB& IF	1:1000/1:100	Santa Cruz Biotechnology (Dallas, TX, USA)
Anti-synaptophysin	Rabbit	sc-7568	WB	1:1000	Santa Cruz Biotechnology (Dallas, TX, USA)
Anti-syntaxin antibody	Mouse	sc-12,736	WB	1:1000	Santa Cruz Biotechnology (Dallas, TX, USA)
Anti-Iba-1 antibody	Mouse	sc-32725	WB	1:1000	Santa Cruz Biotechnology (Dallas, TX, USA)
Anti-GFAP antibody	Mouse	sc-33673	WB& IF	1:1000/1:100	Santa Cruz Biotechnology (Dallas, TX, USA)
Anti-HO1 antibody	Mouse	sc-136961	WB		Santa Cruz Biotechnology (Dallas, TX, USA)
Anti-Nrf2 antibody	Mouse	sc-722	WB& IF	1:1000/1:100	Santa Cruz Biotechnology (Dallas, TX, USA)
Anti-IL-1βeta antibody	Mouse	sc-7884	WB& IF	1:1000/1:100	Santa Cruz Biotechnology (Dallas, TX, USA)
Anti-cytochrome C	Mouse	sc-13156	WB& IF	1:1000/1:100	Santa Cruz Biotechnology (Dallas, TX, USA)
Anti-PARP-1 Antibody	Mouse	sc-56196	WB	1:1000	Santa Cruz Biotechnology (Dallas, TX, USA)
TNF-α antibody	Mouse	sc-52746	WB	1:1000	Santa Cruz Biotechnology (Dallas, TX, USA)

### Methodology of the molecular docking study

We performed a molecular docking study with MOE (Molecular Operating Environment) software to predict the binding mode of Gly in the binding pocket of the JNK protein (PDB ID 3V6S chain A) [29]. The three-dimensional structure of Gly was generated with the builder tool of MOE. Next, the energy was minimized by using the default parameters (gradient: 0.1, Force Field: MMFF94X) after the generated structure was 3D protonated. For further evaluation of molecular docking, the prepared compound was saved as a mdb (Molecular Data Base) file. The 3D structure of the target protein JNK was downloaded from a protein databank. The downloaded structure of the target protein JNK was opened in MOE, water molecules and the B chain were removed, and the JNK protein was 3D protonated. The energy of JNK protein was minimized for protein stability by using the default parameters in MOE. The default parameters, i.e., Placement: Triangle Matcher, Rescoring 1: London dG, Refinement: Force field, Rescoring 2: GBVI/WSA, were used for docking studies. Ten conformations were formed, and on the basis of docking score, the top ranked conformation was selected for further analysis.

### Fluoro-Jade B staining

Fluoro-Jade B staining was performed as previously described [30]. Slides containing brain tissues were air-dried overnight. Following washes with PBS (2 × 10 min), the slides were immersed in 80% ethanol and 1% sodium hydroxide (NaOH) for 5 min. The slides were kept with ethanol (70% v*/*v) before being washed with distilled water for 2–3 min. Next, the slides were washed in distilled water for 2–3 min. Then, the slides were incubated in freshly prepared potassium permanganate (KMNO4, 0.06% w/v) for 10 min with gently shaking before being rinsed with distilled water. Next, the slides were dipped in Fluoro-Jade B (FJB) solution (0.01% v/v) containing 0.1% acetic acid and 0.01% Fluoro-Jade B for 20 min. After this, the slides were rinsed with distilled water, and DAPI was applied after they were dried (placing in an incubator for 10 min). The slides were mounted with glass coverslips using DPX nonfluorescent mounting medium. Representative images were captured using the FITC filter on a confocal laser scanning microscope (FV 1000, Olympus, Japan). The results were analyzed using the computer-based ImageJ program.

### Cresyl violet (Nissl) staining

Nissl staining (cresyl violet) staining was performed to determine the degree of neuronal loss as previously described [31]. Tissue sections (14 μm thick) from the experimental mice were washed with PBS (0.01 M, 2 × 10 min). Cresyl violet solution was prepared by dissolving cresyl violet acetate (Sigma) in distilled water (containing a few drops of glacial acetic acid) at a concentration of 0.5% (w/v). The brain sections were stained with cresyl violet solution for approximately 10 min; rinsed with distilled water; dehydrated in 70% alcohol (for 5 min), 95% alcohol (for 5 min), and 100% alcohol (for 5 min); placed in xylene for 5 min; and covered with glass coverslips using nonfluorescent mounting medium, and images were taken with a fluorescent light microscope. The results were analyzed using computer-based ImageJ software, and GraphPad Prism 6 software was used to generate histograms.

### Data and statistical analyses

In brief, ImageJ software was used to measure both the western blot data (density in arbitrary units (A.U.s) and the morphological data (integrated density in A.U.). The density values are expressed as the mean ± standard error mean (SEM) of triplicate wells (for in vitro experiments) and of 8 mice/group (for in vivo experiments) and are representative of three independent experiments. To generate graphs, we used GraphPad Prism 6 software (San Diego, CA, USA), and one-way ANOVA followed by Tukey’s post hoc test was used for statistical analysis of differences among the experimental groups. Differences between groups were considered significant at *p* < 0.05 (significance: *^#abcd^*p* ≤ 0.05; **^##^*p* ≤ 0.01; ***^###^*p* ≤ 0.001]. *^a^ indicates a significant difference from the vehicle-treated control group, while ^#bcd^ indicates a significant difference from the d-gal-treated groups.

## Results

### Glycine treatment attenuated d-galactose-induced cytotoxicity and ROS/LPO levels in HT-22 cells

**Fig. 1:**
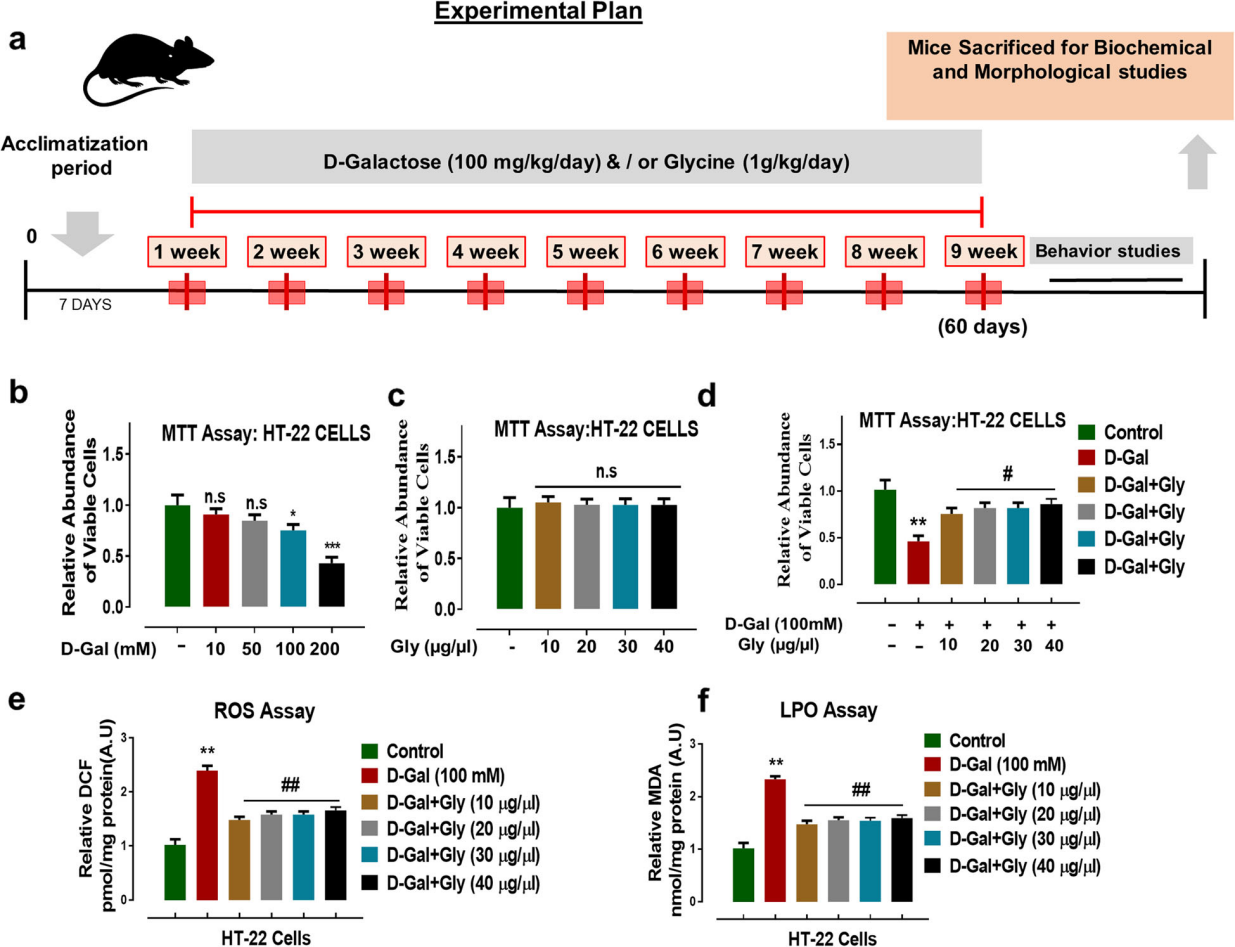
Experimental design, cytoprotective and antioxidant effects of glycine in d-galactose-treated in HT22 cells. **a** Experimental design of experimental work for the drug treatment in C57BL/6 mice and behavioral analysis. **b**–**d** Results of MTT (cell viability) assay and their relative histogram in neuronal HT22 cells. Number of experiment (*N* = 3). **e**, **f** Representative histograms of reactive oxygen species (ROS measured as DCF level) and lipid peroxidation (LPO measured MDA level) assays of Gly against D-gal in HT22 cells. The data are shown as the mean ± SEM of triplicate wells for in vitro experiments are representative of three independent experiments. Significance: Asterisk (*) sign indicated significant difference from the normal saline-treated control group; hash (#) sign indicated significant difference from d-gal-treated group. Significance: *P ≤ 0.05, **P ≤ 0.01; ***P ≤ 0.001; #*P* ≤ 0.05, ##*P* ≤ 0.01. *n*.*s* no significance

The effect of d-gal and/or Gly on cell viability was evaluated by determining the viability of treated mouse hippocampal neuronal HT22 cells by the MTT assay according to the manufacturer's instructions (Sigma Aldrich). To measure the effects of d-gal and Gly on cell viability, HT22 cells were treated with different concentrations of d-gal (10, 50, 100, or 200 mM) and Gly (10, 20, 30, or 40 μg/μL) for 24 h respectively. The MTT results indicated that d-gal treatment significantly reduced cell viability in a dose-dependent manner after 24 h (Fig. 1b). However, Gly was not toxic to the HT22 cells at any of the tested concentrations. No significant difference was found in viability between control cells and Gly-treated cells, indicating that Gly was not toxic to HT22 cells at all treated concentrations (Fig. 1c). Importantly, d-gal (100 mM) + Gly (10, 20, 30, 40 μg/μL) cotreatment significantly increased viability/cell survival and protected HT22 cells against d-gal-induced cytotoxicity (Fig. 1d).

Furthermore, to determine the antioxidant effect of Gly against d-gal-induced oxidative stress, we performed reactive oxygen species (ROS) and lipid peroxidation (LPO) assays in vitro. The in vitro assays indicated a significant increase in DCF (ROS levels) and LPO (MDA levels), suggesting that compared to control treatment, d-gal alone increased oxidative stress in HT22 cells (Fig. 1e, f). However, d-gal + Gly cotreatment significantly reduced the elevated ROS and MDA levels, as shown by the DCF and LPO assays (Fig. 1e, f). These findings indicate that Gly mitigates oxidative stress induced by d-gal in HT22 cells.

### Glycine treatment reduced ROS/MDA levels and increased Nrf-2/HO-1 levels in the brains of d-galactose-treated mice

**Fig. 2:**
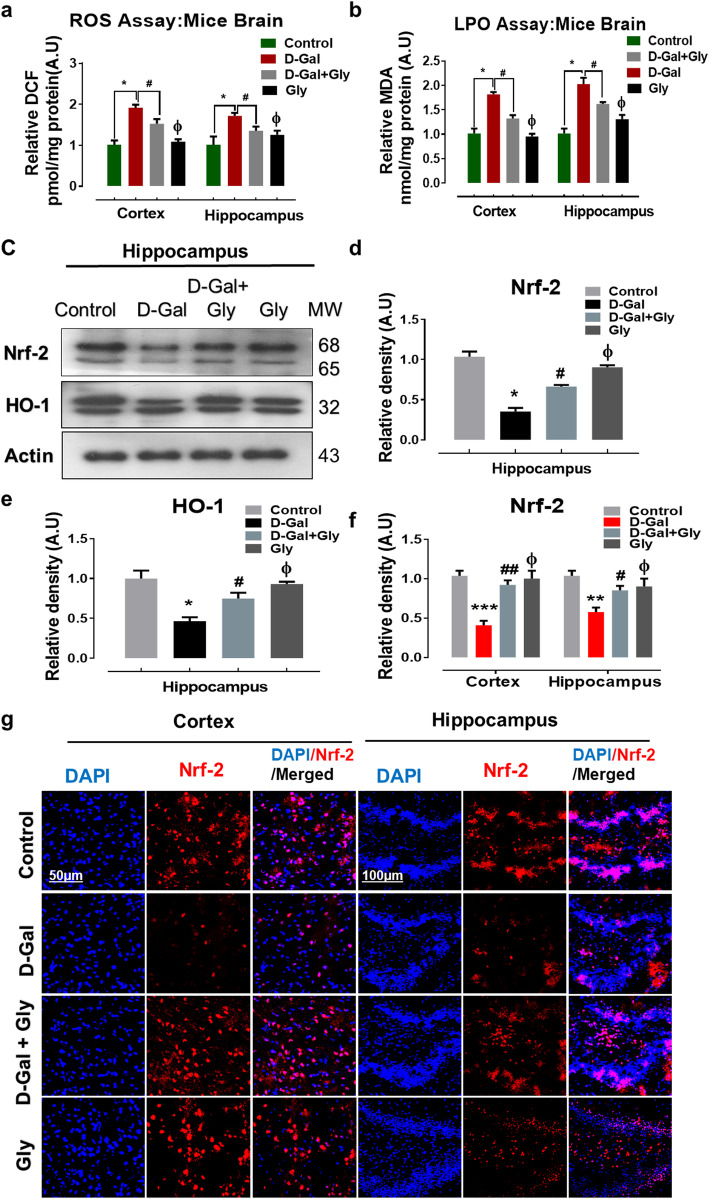
Glycine treatment inhibited d-galactose-induced oxidative stress and ameliorates ROS/LPO production in mice brain. **a**, **b** Representative histograms of reactive oxygen species (ROS measured as DCF level) and lipid peroxidation (LPO measured as MDA level) assays of Gly against d-galactose in the cortex and hippocampus of mice brain. **c**–**e** Western blot analysis representing the expression level of Nrf2 and HO1 proteins in the hippocampus of the experimental groups. The cropped bands were quantified using ImageJ software, and the differences are represented in the histogram. The density values are expressed in arbitrary units (A.U.) as the mean ± SEM for the respective indicated protein. An anti-β-actin antibody was used as a loading control. *n* = 8 mice/group, and the number of experiments performed *N* = 3. **g** The immunofluorescence images represented the immunoreactivity of Nrf2 proteins (Red, TRITC; Blue, DAPI) in the cortex and hippocampus regions of mice brain along with their relative histograms, respectively. The relative integrated density values are represented in arbitrary units (A.U) as the means (± S.E.M) for the respective indicated proteins. DAPI (blue) was used for nucleus staining. *n* = 8 mice/group, and the number of experiments = 3. Magnification × 40. Scale bar; 50 μm = cortices; DG hippocampal regions =100 μm. Asterisk (*) sign indicated significant difference from the normal saline treated group; hash (#) sign indicated significant difference from d-gal-treated group; while the phi (Φ) sign indicated no significance from normal saline-treated control group. Significance: *P ≤ 0.05, **P ≤ 0.01; ***P ≤ 0.001; #*P* ≤ 0.05, ##*P* ≤ 0.01

Previous studies have shown that d-gal is involved in oxidative stress-mediated neurodegeneration [1, 23]. To determine the antioxidant effect of Gly against d-gal-induced oxidative stress in the mouse brain, we performed reactive oxygen species (ROS) and lipid peroxidation (LPO) assays in vivo. Our results demonstrated that d-gal treatment considerably elevated ROS generation in the brains of d-gal-treated mice compared with the brains of normal saline-treated (control) mice. Conversely, Gly significantly reduced elevated ROS levels in both studied regions (the cortex and hippocampus) of the mouse brain. Similarly, Gly significantly reduced the increased LPO level induced by d-gal in the cortex and hippocampus of the mouse brain (Fig. 2a, b).

**Fig. 3:**
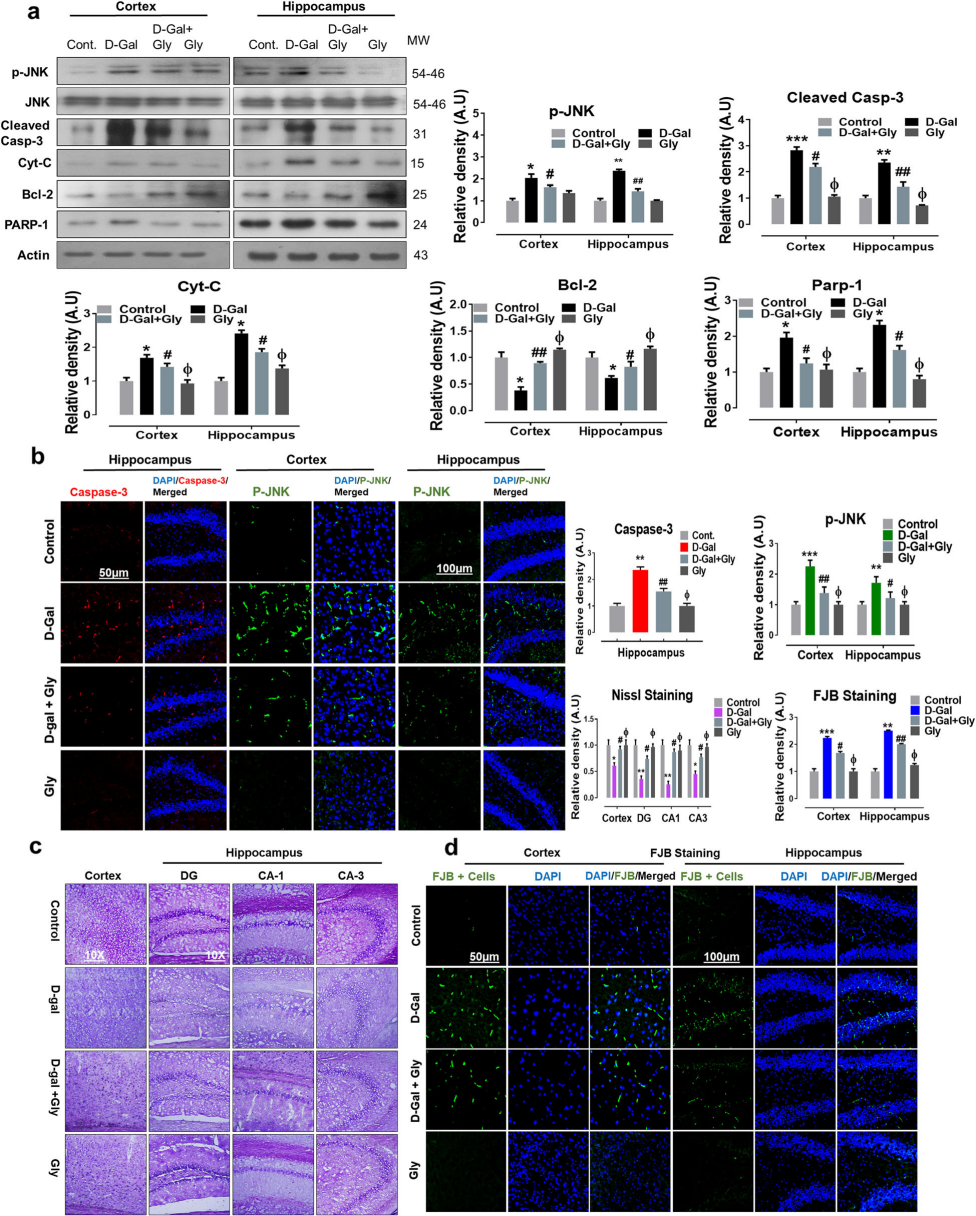
Glycine treatment inhibited d-galactose-induced elevated p-JNK and apoptotic cell death in mice brain. **a** Representative western blot analysis of stress kinase phosphorylated (p-JNK), cleaved caspase-3, cytochrome c (Cyt. C), Bcl-2 (B-cell lymphoma 2), and PARP-1 (poly-ADP-ribosyltransferase) proteins expression levels in both cortex and hippocampus regions of mice brain. The cropped bands were quantified using ImageJ software, and the differences are represented in the histogram. The density values are expressed in arbitrary units (A.U.) as the mean ± SEM for the respective indicated protein. An anti-β-actin antibody was used as a loading control. *n* = 8 mice/group, and the number of experiments performed *N* = 3. **b** Representative immunofluorescence results of caspase-3 (red; in hippocampus) and activated p-JNK proteins (green; in cortex and hippocampus) of the experimental mice group and **c**, **d** Nissl (cortex, DG, CA1, and CA3 regions) FJB staining (green, FITC; Blue) in cortex and hippocampus regions of experimental mice brain. The relative integrated density values are represented in arbitrary units (A.U) as the means (± S.E.M) for the respective indicated proteins. DAPI (blue) was used for nucleus staining. *n* = 8 mice/group, and the number of experiments = 3. Magnification × 40. Scale bar; 50 μm = cortices; DG hippocampal regions =100 μm. Asterisk (*) sign indicated significant difference from the normal saline treated group; hash (#) sign indicated significant difference from d-gal-treated group; while the phi (Φ) sign indicated no significance from normal saline-treated control group. Significance: *P ≤ 0.05, **P ≤ 0.01; ***P ≤ 0.001; #*P* ≤ 0.05, ##*P* ≤ 0.01

Furthermore, we checked the expression levels of antioxidant proteins, including nuclear factor erythroid 2-related factor 2 (Nrf2) and heme oxygenase-1 (HO-1), by Western blotting to further confirm the antioxidant effect of Gly against d-gal in the mouse brain. Our immunoblot results revealed that the expression levels of Nrf-2 and HO-1 were substantially reduced in the brains of d-gal-treated mice compared to the brains of saline-treated mice. However, the expression level of the aforementioned antioxidant proteins was upregulated in the brains of d-gal + Gly-treated mice brain in comparison with the brains of d-gal-treated mice (Fig. 2c–e). Immunofluorescence analysis confirmed the Western blotting results for Nrf2; Nrf2 reactivity was significantly increased in both the cortex and hippocampus in the brains of d-gal + Gly-treated mice in comparison with d-gal-treated mice group (Fig. 3f, g). Additionally, both immunoblot and immunofluorescence analysis indicated that Gly was not toxic to the normal mice and showed no significant difference between normal saline-treated mice and Gly alone-treated mice (Fig. 2). Taken together, these findings indicate that through its antioxidant activities, Gly reverses d-gal-induced oxidative stress in the mouse brain.

### Glycine treatment inhibited protein activation of p-JNK and apoptotic neurodegeneration mediated by d-galactose-induced oxidative stress in the mouse brain

Studies have reported that d-gal-mediated elevations of p-JNK levels initiate the mitochondrial apoptotic pathway, leading to neurodegeneration [32–35]. Therefore, consistent with these reports, we analyzed the effect of Gly against protein activation of p-JNK and its downstream neuroapoptotic markers mediated by d-gal-induced oxidative stress in the mouse brain. Our immunoblot results indicated that compared to saline treatment, d-gal treatment significantly increased the expression of p-JNK in both the cortex and hippocampus of the mouse brain. However, compared with d-gal alone, d-gal + Gly cotreatment markedly reduced the elevated expression of p-JNK (Fig. 3a). Additionally, immunoblot analysis showed that Gly alone treatment was not toxic to normal mice and revealed no significant difference between the brains of normal saline-treated (control) mice and those of Gly alone-treated mice. Similarly, early studies reported that activation of p-JNK by chronic treatment with d-gal has a direct impact on cytochrome C (Cyt C) release, which further activates caspases to mediate neuroapoptosis and neurodegeneration [36]. Therefore, to determine whether Gly reverses d-gal-induced neuronal apoptosis, we performed western blotting to examine the expression of various proapoptotic and antiapoptotic markers. Consistently, our results indicated that d-gal treatment significantly upregulated the protein expression level of cleaved caspase-3, cytochrome c (Cytc), and poly (ADP-ribose) polymerase 1 (PARP-1) while markedly downregulating the protein expression level of B cell lymphoma 2 (Bcl-2) in both the cortex and hippocampal of the brains of d-gal-treated mice. Conversely, d-gal + Gly cotreatment significantly reduced the elevated expression levels of cleaved caspase-3, Cyt C, and PARP-1 while upregulating the protein expression level of Bcl-2 in both indicated regions of the mouse brain (Fig. 3a). Additionally, our immunofluorescence analysis also indicated significantly higher expression of p-JNK (in the cortex and hippocampus) and caspase-3 (in the hippocampus) in the brains of d-gal-treated mice than those of normal saline-treated (control) mice. However, d-gal + Gly cotreatment significantly reversed the increase in immunofluorescence reactivity of p-JNK and caspase-3 in both indicated regions of the mouse brain (Fig. 3b).

Furthermore, FJB and Nissl staining indicated that d-gal + Gly cotreatment reversed the effects of d-gal by significantly reducing the number of FJB-positive (+ve) neuronal cells (in the cortex and hippocampus) and increasing the number of surviving neurons (in the cortex, DG, CA-1, and CA-3 regions of the hippocampus) in comparison with those in the brains of d-gal-treated mice (Fig. 3c, d). Taken together, these results indicate that Gly is effective in preventing protein activation of p-JNK and its downstream neuroapoptotic markers mediated by d-gal-induced oxidative stress in the mouse brain.

### Glycine treatment ameliorated d-galactose-mediated neuroinflammation and glial cell activation in the brains of d-galactose-treated mice

**Fig. 4:**
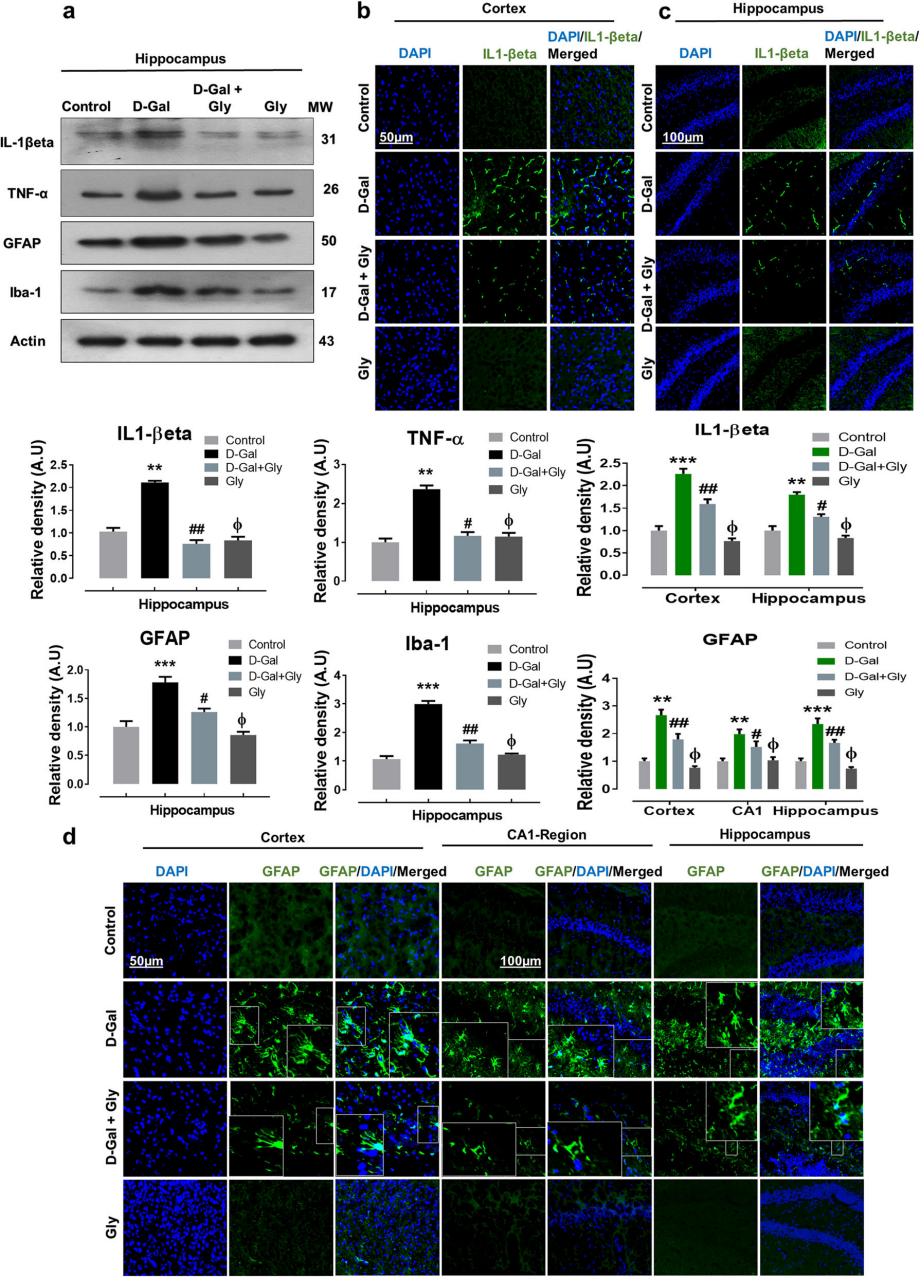
Gly inhibited d-galactose-induced activation of inflammatory proteins in the hippocampus of mice brain. **a** The Western blot analysis of tumor necrosis factor alpha (TNF-α), interleukin-1 βeta (IL-1βeta), glial fibrillary acidic protein (GFAP), and ionized calcium binding adaptor molecule 1 (Iba1) protein expression level in the hippocampus of mice The cropped bands were quantified using ImageJ software, and the differences are represented in the histogram. The density values are expressed in arbitrary units (A.U.) as the mean ± SEM for the respective indicated protein. An anti-β-actin antibody was used as a loading control. *n* = 8 mice/group, and the number of experiments performed *N* = 3. **b**, **c** The immunofluorescence images represent the immunoreactivity of IL-1βeta (green, FITC; Blue, DAPI) in cortex and hippocampus of mice (**d**) The immunofluorescence images represent the immunoreactivity of GFAP (green, FITC; blue, DAPI) in cortex and hippocampus (CA-1 and DG regions) of mice The relative integrated density values are represented in arbitrary units (A.U) as the means (± S.E.M) for the respective indicated proteins. DAPI (blue) was used for nucleus staining. *n* = 8 mice/group, and the number of experiments = 3. Magnification × 40. Scale bar; 50 μm = cortices; DG hippocampal regions =100 μm. Asterisk (*) sign indicated significant difference from the normal saline-treated group; hash (#) sign indicated significant difference from d-gal-treated group; while the phi (Φ) sign indicated no significance from normal saline-treated control group. Significance: *P ≤ 0.05, **P ≤ 0.01; ***P ≤ 0.001; #*P* ≤ 0.05, ##*P* ≤ 0.01.

Accumulating evidence suggests that d-gal-induced p-JNK activation results in the activation and accumulation of various inflammatory mediators [1, 37]. Therefore, we detected various inflammatory mediators, including interleukin 1 beta (IL-1βeta) and tumor necrosis factor-alpha (TNF-α), by Western blotting and immunofluorescence analysis. We found significantly higher protein expression of TNF-α and IL-βeta in the hippocampi of d-gal-treated mice than in the hippocampi of saline-treated (control) mice through Western blot analysis. However, d-gal + Gly cotreatment reversed the effect of d-gal and compared to d-gal alone, significantly reduced the increased expression of IL-1βeta and TNF-α in the hippocampus (Fig. 4a). Additionally, IL-1βeta immunofluorescence reactivity was significantly increased in both the cortex and hippocampus in the brains of d-gal alone-treated mice compared to those of saline-treated (control) mice. Interestingly, compared to d-gal alone, d-gal + Gly cotreatment significantly reduced the immunofluorescence reactivity of IL-1βeta in both the cortex and hippocampus (Fig. 4b, c).

Accumulative evidence has shown that astrocytosis and microgliosis increase with age and are higher in mouse model of d-gal-induced aging compared to control mice [5, 38]. To analyze the effect of Gly on the expression of GFAP and Iba-1, we performed immunoblotting and immunofluorescence. We found significant increases in the protein expression of GFAP and Iba-1 in the hippocampus in the brains of d-gal-treated mice compared to those of saline-treated (control) mice, as shown by Western blot analysis (Fig. 4a). However, compared to d-gal alone, d-gal + Gly cotreatment significantly reduced the elevated expression of GFAP and Iba-1 in the hippocampal region, indicating potent inhibition of microgliosis and astrocytosis (Fig. 4a). Likewise, our confocal microscopy results showed that GFAP immunofluorescence reactivity was significantly increased in the cortex and hippocampus (the CA1 region and dentate gyrus (DG)) in the brains of d-gal-treated mice compared to those of saline-treated (control) mice. Interestingly, compared to d-gal alone, d-gal + Gly cotreatment significantly reduced GFAP immunofluorescence in the cortex and hippocampus (the CA1 region and dentate gyrus (DG)) (Fig. 4d). Notably, both immunoblot and immunofluorescence analysis indicated that Gly was not toxic to the control mice and showed no significance difference between normal saline-treated mice and Gly alone-treated mice (Fig. 4). These findings indicate that Gly limits d-gal-induced p-JNK-mediated neuroinflammation in the hippocampus of the mouse brains.

### Glycine treatment improved synaptic dysfunction and enhanced learning, memory, and spontaneous alteration behavior in the brains of mice with d-galactose-induced memory impairment

**Fig. 5:**
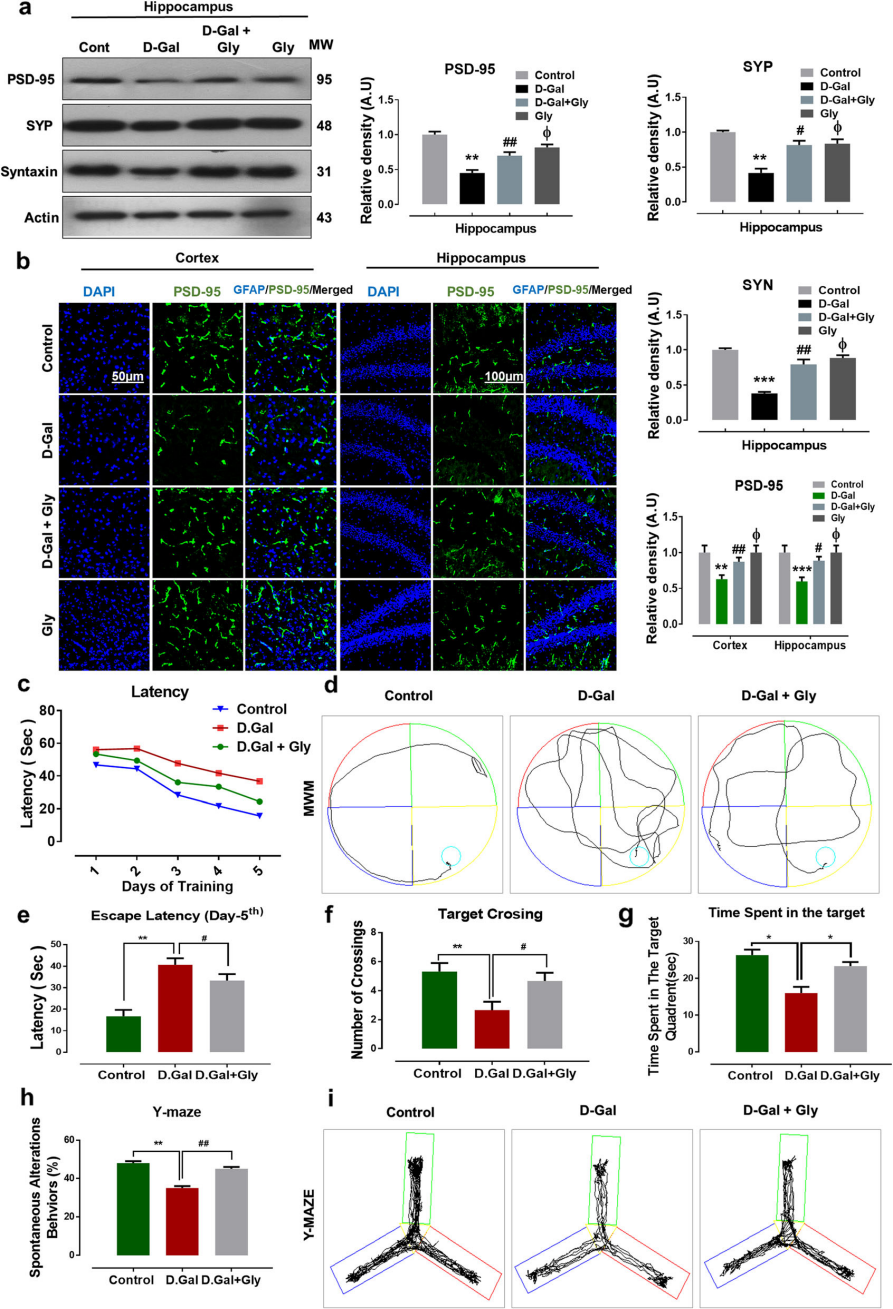
Gly inhibited d-galactose-induced synaptic and memory dysfunction in C57BL/6 mice brain. **a** Western blot analysis of presynaptic proteins including synaptophysin (SYP), syntaxin (SYN), and postsynaptic density proteins (PSD95) in the hippocampus of different experimental groups The cropped bands were quantified using ImageJ software, and the differences are represented in the histogram. The density values are expressed in arbitrary units (A.U.) as the mean ± SEM for the respective indicated protein. An anti-β-actin antibody was used as a loading control. *n* = 8 mice/group, and the number of experiments performed *N* = 3. **b** The immunofluorescence images represented the immunoreactivity of PSD95 (green, FITC; blue, DAPI) in cortex and hippocampus of mice brain; along with their relative histograms, respectively. The relative integrated density values are represented in arbitrary units (A.U) as the means (± S.E.M) for the respective indicated proteins. DAPI (blue) was used for nucleus staining. *n* = 8 mice/group, and the number of experiments performed *N* = 3. Magnification × 40. Scale bar; 50 μm = cortices; DG hippocampal regions = 100 μm. **c** Mean escape latency in seconds to reach the hidden platform during training (5 days) along **d** with representative trajectories; **f** number of target crossings; **g** the time spent in the target quadrant; **h** Y-maze analysis represented spontaneous alteration behaviors along with its representative trajectories of mice. For behavioral study, the number of mice (*n* = 16) per experimental group was used. Asterisk (*) sign indicated significant difference from the normal saline treated group; hash (#) sign indicated significant difference from d-gal-treated group; while the phi (Φ) sign indicated no significance from normal saline-treated group

Several studies have reported altered expression of synaptic proteins or genes in d-gal-treated animals [23, 39]. To investigate the neurotoxic effect of d-gal and the neuroprotective effects of Gly on synaptic proteins, we performed Western blotting to analyze the expression of pre- and postsynaptic proteins. Our immunoblotting results revealed that memory-related presynaptic proteins, including synaptophysin (Syp), syntaxin (Syn), and a postsynaptic density protein (PSD95), were markedly decreased in the brains of d-gal-treated mice compared with those of saline-treated control mice. Interestingly, compared with d-gal alone, d-gal + Gly cotreatment significantly increased the expression levels of PSD95, SYP, and Syn in the hippocampus (Fig. 5a). Furthermore, our confocal microscopy results also suggested that PSD95 immunofluorescence reactivity was significantly decreased in the cortex and hippocampus in the brains of d-gal-treated mice compared with those of saline-treated (control) mice. However, compared with d-gal alone, d-gal + Gly cotreatment significantly increased the immunofluorescence reactivity of PSD95 in both regions (the cortex and hippocampus) (Fig. 5b). Additionally, both immunoblot and immunofluorescence analysis indicated that Gly was not toxic to the control mice and showed no significance difference between normal saline-treated mice and Gly alone-treated mice (Fig. 5).

Furthermore, to analyze the effect of d-gal and Gly on mouse behavior and memory, we performed the Morris water maze (MWM) and Y-maze tests as previously described [5]. In the MWM test, the mean latency (to find the hidden platform) gradually decreased over training days in all mouse groups, except the d-gal-treated group, which exhibited a longer latency than the saline-treated (control) group, indicating impaired spatial learning and memory ability. However, compared with d-gal alone, d-gal + Gly cotreatment significantly shortened the latency to reach the platform (Fig. 5c–e). Similarly, after completion of the trial session, we removed the hidden platform and performed a probe test. We found that the number of platform crossings was significantly increased in the d-gal + Gly-cotreated group compared with the d-gal-treated group (Fig. 5f). In addition, we found that the d-gal + Gly-cotreated mice spent more time in the target quadrant than the d-gal-treated mice (Fig. 5g), showing that Gly reduced d-gal-induced memory impairment.

Following the MWM test, we performed the Y-maze test to analyze spatial working memory based on the spontaneous alteration behavior percentage (%). A higher percentage (%) of spontaneous alteration behavior was indicative of improved cognitive performance. We found that the d-gal-treated mice exhibited a significantly lower percentage (%) of spontaneous alterations than the saline-treated (control) mice, indicating impaired working memory. However, compared to the d-gal-treated mice, the d-gal + Gly-cotreated mice showed a significant increase in spontaneous alteration behavior (%), indicating that Gly attenuated short-term memory deficits in the d-gal-treated mice (Fig. 5h, i).

### Glycine treatment confers neuroprotection against d-galactose-induced neurotoxicity via downregulation of c-Jun N-terminal kinase in HT22 cells

**Fig. 6:**
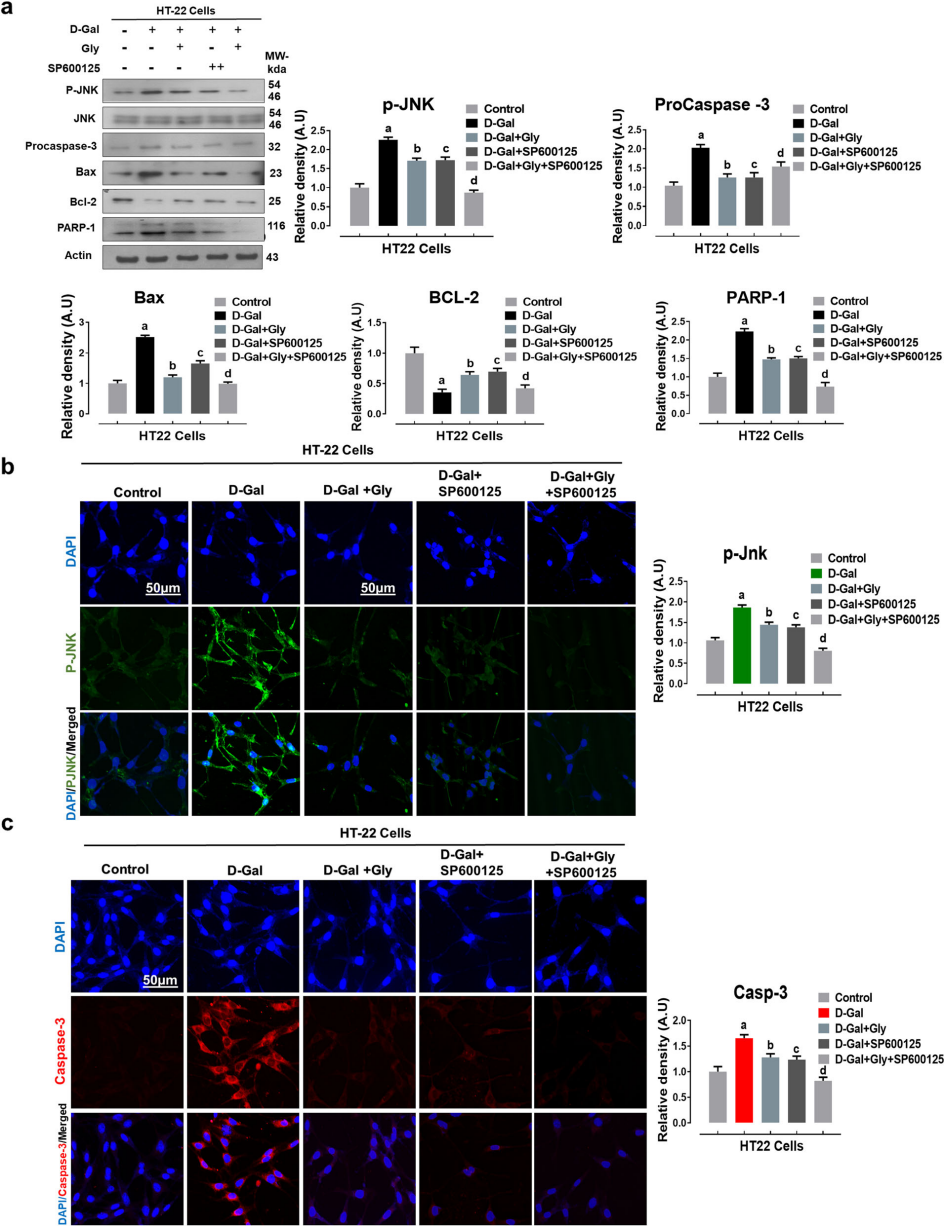
Glycine treatment reduced d-galactose-mediated elevated p-JNK-dependent neuroapoptosis in HT22 cells lines. **a** Representative western blot analysis of activated phosphorylated (p-JNK), procaspase-3, BCL2-associated X protein (Bax), Bcl-2 (B-cell lymphoma 2), and poly [ADP-ribose] polymerase 1 (PARP-1) proteins expression levels with or without JNK inhibitor (SP600125) in the HT22 cell line. The cropped bands were quantified using ImageJ software, and the differences are represented in the histogram. The density values are expressed in arbitrary units (A.U.) as the mean ± SEM for the respective indicated protein. An anti-β-actin antibody was used as a loading control. Number of experiments performed *N* = 3. **b**, **c** Immunofluorescence images of activated p-JNK (green) and caspase-3 (red) proteins along with their relative histograms after drug treatment with d-gal (100 mM), Gly (20 μg/μl), and SP600125 (20 μM) treatment in HT22 cell line for 24 h. The relative integrated density values are represented in arbitrary units (A.U) as the means (± S.E.M) for the respective indicated proteins. DAPI (blue) was used for nucleus staining. The data are expressed as the mean ± SEM. Magnification × 40. Scale bar; 50 μm. ^a^ Significantly different from the control group while ^bcd^ significantly different from the d-gal-treated groups. Significance: ^a, b, c, d^*P* < 0.05

After analyzing the aforementioned antiapoptotic and anti-inflammatory effects of Gly against d-gal in the mouse brain, we attempted to study the exact mechanism by which Gly confers neuroprotection. As reported in earlier studies, aberrant activation of the p-JNK protein in the hippocampus plays critical roles in mediating neuronal apoptosis [40]. Therefore, we hypothesized that the in vivo neuroprotective effect of Gly might be due to inhibition of d-gal-induced elevation of p-JNK levels. Thus, we examined the protein expression level of activated phosphorylated JNK (p-JNK) and its downstream signaling molecules in HT22 cells in vitro. Interestingly, consistent with our in vivo findings, our immunoblotting results revealed that compared with control treatment, d-gal alone significantly upregulated the protein expression levels of p-JNK and its downstream signaling molecules, including procaspase-3, Bax, and cleaved PARP-1, while significantly downregulating the protein expression level of an antiapoptotic protein (Bcl-2) in HT22 cells. However, compared to d-gal alone, d-gal + Gly cotreatment significantly downregulated the elevated protein expression levels of p-JNK, pro-caspase-3, Bax, and PARP-1 but upregulated the expression level of an antiapoptotic protein (Bcl-2) in neuronal HT22 cells (Fig. 6a). Additionally, the immunofluorescence reactivity of p-JNK and its downstream target caspase-3 was markedly decreased in Gly-treated HT22 cells compared to d-gal-treated HT22 cells.

**Fig. 7:**
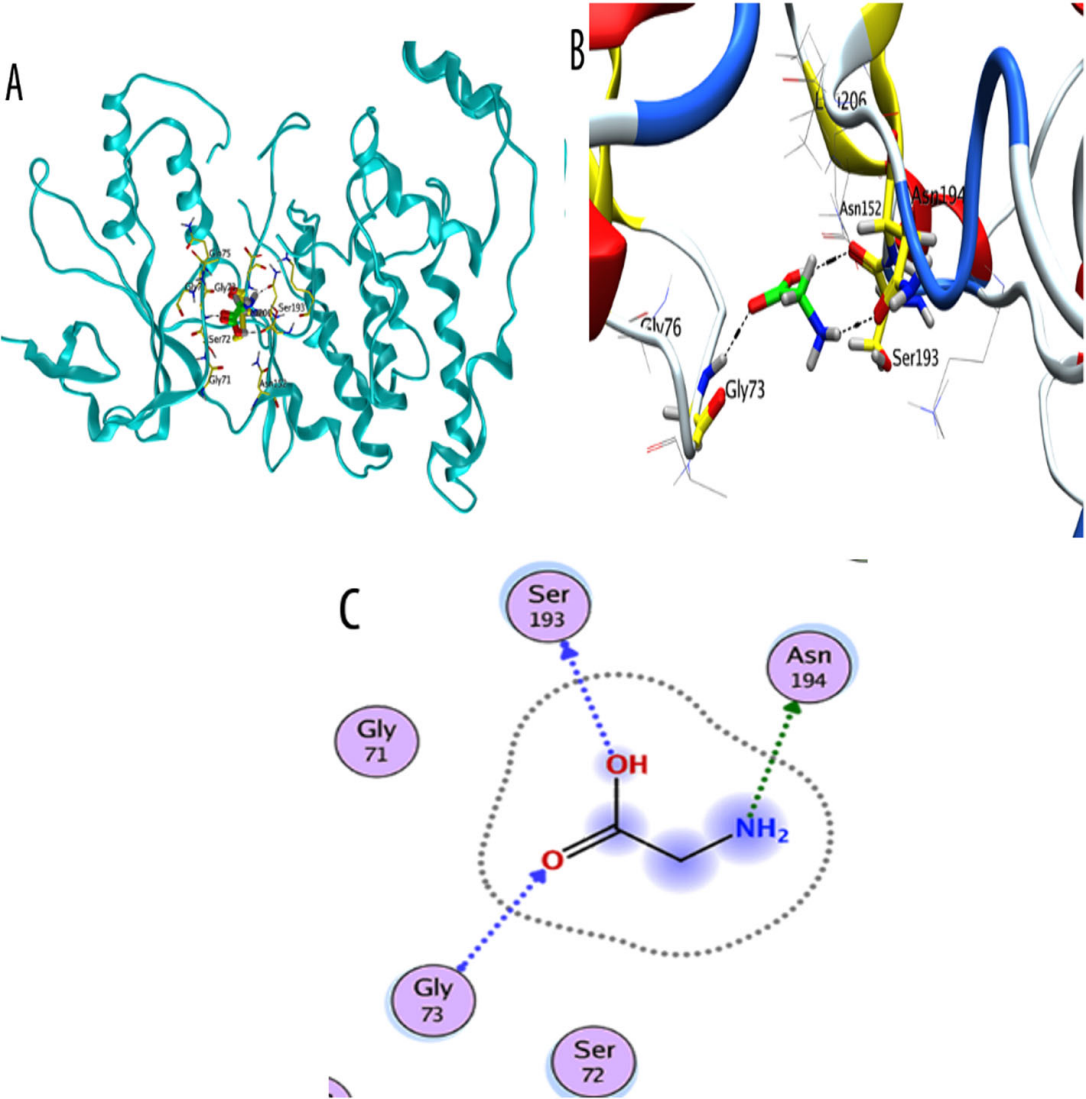
Docking conformation of glycine on JNK protein (PDB ID 3v6s chain A). **b** Binding mode of glycine as inhibitor of JNK. Ligand is shown in bright green color. **b** 3D binding mode of glycine with active site residues Gly73, Ser193, and Asn194 form hydrogen acceptor bonds with (C=O), OH, and NH_2_ group of glycine respectively. **c** 2D binding mode of glycine as inhibitor of JNK showing H-donor and acceptor interactions

To determine whether the neuroprotective effect of Gly against d-gal-induced neurotoxicity is JNK-dependent, we used a pharmacological JNK-specific inhibitor (SP600125) in HT22 cells*.* Interestingly, our in vitro immunoblotting and immunofluorescence results indicated that compared to other treatments, d-gal + Gly + SP600125 cotreatment markedly downregulated the protein expression and immunoreactivity of p-JNK levels and procaspase-3, which had been elevated by d-gal, reduced the protein expression of downstream signaling molecules of p-JNK (Bax and cleaved PARP-1), and upregulated the protein expression level of Bcl-2 in HT22 cells (Fig. 6a, b). In addition to these (wet laboratory) results, our molecular docking (dry laboratory ) studies (shown in Fig. 7) revealed stable Gly-JNK protein interactions in which Gly interacted with multiple key residues of the target protein JNK to inhibit its function. Collectively, these results indicate that Gly reverses d-gal-induced elevation of p-JNK levels and downstream signaling in a JNK-dependent manner in HT22 cells in vitro.

### Molecular docking study: confirmation and interactions of glycine with the JNK protein

We performed a molecular docking study to confirm the mechanism underlying the interaction between Gly and JNK. Among the different conformations, we observed the most favorable docking pose involves Gly inside the binding pocket of JNK in the proper orientation. The binding site comprises both hydrophobic and hydrophilic amino acids. The hydrophobic part resides include Ile70, Val78, Ala91, Ile124, Met146 and 149, Ala151, Val196, and Leu206, while the hydrophilic amino acids are Gly71, 73, Ser72, Gln75, Gly76, Lys93, Glu147, Asp150, Cys154, Gln155, 158, Ser193, Asn194, and Asn255. Analysis of the binding mode of the most favorable docking conformation revealed that the compound interacts well with hydrophilic amino acids (Gly73, Ser193, Asn194) over the binding cavity through OH, NH_2_, and (C=O) groups by forming hydrogen bonds (Table 1). Figure 7 shows a 3-D depiction of the interactions associated with the favorable conformation. Thus, the binding ability of Gly was very strong in terms of binding affinity (− 3.81 Kcal/mol), solvation energy (− 18.63 Kcal/mol), and docking score (− 15.5809 Kcal/mol). These values indicated good fitness of Gly in the binding pocket of the target protein and a stable Gly-JNK protein interaction. These theoretical results are consistent with the fact that Gly showed much stronger activity than other amino acids by engaging in multiple interactions with the key residues of the target protein JNK.

## Discussion

Aging is one of the major factors in numerous chronic diseases, including diseases of the CNS [2, 3]. d-gal is a well-established agent for modeling brain aging and triggers neuronal death by upregulating JNK levels in animal models [5, 10, 23]. Herein, we identified the molecular mechanism underlying the neuroprotective effect of Gly (the smallest and simplest amino acid) against d-gal-mediated neurodegeneration and memory impairment. Our results indicated that d-gal induced oxidative stress-mediated neurodegeneration primarily by upregulating p-JNK levels. However, d-gal + Gly cotreatment reversed the neurotoxic effects of d-gal by downregulating d-gal-induced elevated p-JNK levels. Notably, both the molecular docking study and the in vitro study (in which neuronal HT22 cells were treated with or without a p-JNK-specific inhibitor (SP600125)) further support our in vivo findings that Gly binds to the p-JNK protein and inhibits its function. Therefore, we suggest that Gly-mediated deactivation of the JNK signaling pathway underlies the neuroprotective effect of Gly, which reverses d-gal-induced oxidative stress, apoptotic neurodegeneration, neuroinflammation, synaptic dysfunction, and memory impairment.

Oxidative stress has been suggested to be a common etiology of various CNS disorders [41]. Recent reports have demonstrated that chronic administration of d-gal (i/p for 8 weeks) increases oxidative stress and weakens antioxidant enzymes, which leads to aging and age-related memory impairment [23, 42]. Nrf2, a master endogenous antioxidant gene, is a stress-responsive transcription factor that is activated in response to oxidative stress and in turn activates other endogenous redox-regulated enzymes (HO-1), to counteract ROS-induced oxidative stress in various diseases, particularly neurodegenerative diseases [41]. In this regard, several studies have reported that d-gal-induced oxidative stress downregulates the protein expression of Nrf2 and HO-1 [43, 44]. Accordingly, our ROS and LPO assays indicated that Gly treatment significantly inhibited ROS and LPO levels in both d-gal-treated HT22 cells and the mouse brain. Moreover, we also found that Gly treatment reversed the d-gal effect by significantly increasing the protein expression levels of Nrf2 and HO-1. These findings indicate that Gly enhances the antioxidant response and limits d-gal-induced oxidative stress.

The MAPK pathway, a conserved signal-transduction pathway, plays a vital role in modulating many physiological processes, including metabolism, gene expression, differentiation, mitosis, and apoptosis [45]. In mammalian cells, the JNK/SAPK (C-Jun N-terminal kinase/stress-activated protein kinase) family is a subfamily belonging to the MAPK superfamily [46, 47]. Recent studies have reported that JNK/SAPK are involved and activated in response to oxidative stress-induced insults [48] in numerous human diseases, including chronic inflammation, birth defects, cancer, ischemia/reperfusion injury, and neurodegeneration, particularly Parkinson’s disease and Alzheimer’s disease [49]. Several other studies have shown that oxidative stress elevates p-JNK levels in a model of d-gal-induced aging [1, 50]. Similarly, other studies have shown that JNK can function as a proapoptotic kinase that can induce neuroapoptosis, leading to cell death [11]. Others have reported that elevated p-JNK levels stimulate Cyt C/Caspase activation, leading to activation of the apoptotic signaling pathway [1]. The cytosolic release of cytochrome-C (Cyt C) stimulates the apoptotic protease cascade, which activates caspase-3 and thereby causes neuronal cell death [34]. Furthermore, poly (ADP-ribose) polymerase 1 (PARP-1) activation in response to elevated p-JNK levels is another hallmark of neuronal apoptosis [51]. Conversely, targeting p-JNK with a small-molecule peptide and specific JNK inhibitor (SP600125) alleviates JNK-mediated neuroapoptosis and neurodegeneration [49, 52]. Similarly, knockdown of JNK prevents apoptosis, whereas overexpression of JNK results in apoptosis in cultured sympathetic neurons [11, 53]. Accordingly, our in vitro and in vivo immunoblot results indicated that Gly treatment significantly downregulated the elevated protein expression level of p-JNK and its downstream apoptotic markers, including pro-caspase-3, Bax, and PARP-1, whereas it significantly upregulated the protein expression level of Bcl-2. In addition, the activated p-JNK and caspase-3 immunofluorescence and the FJB and Nissl staining indicated that Gly prevented apoptosis and neurodegeneration in the d-Gal-treated mouse brain. Importantly, we also elucidated the underlying mechanism by demonstrating that Gly prevented D-Gal-induced neurotoxicity in vitro in HT22 cells in a JNK-dependent manner because when JNK was inhibited with a specific inhibitor (SP600125) the d-gal-induced activation of the JNK-dependent apoptotic pathway was significantly reversed. Moreover, to further confirm these results, we performed a molecular docking (dry laboratory) study, which supported our findings that Gly forms a stable interaction with the JNK protein to inhibit its function. In addition to this, others reported that Gly act as co-agonist via *N*-methyl-d-aspartate receptor (NMDAR) to confer neuroprotection [54, 55]. Therefore, our findings indicate an additional/alternative neuroprotective mechanism of Gly against d-gal-induced apoptosis via suppression of the JNK-mediated apoptotic pathway.

Other studies have suggested that JNK, in addition to playing a critical role in mediating apoptotic signaling [56], is an important mediator of microglial activation [57] and neuroinflammation [58]. Chronic neuroinflammation is a potential risk factor for a broad range of age-associated diseases [59]. Additionally, the complex interactions between glial cells (microglia and astrocytes) are involved in acceleration of the neuroinflammatory response in neurodegenerative disorders [60]. Glial fibrillary acidic protein (GFAP) and ionized calcium-binding adaptor molecule (Iba-1) are specific markers of the activation of astrocytes and microglia, respectively [50]. Several lines of evidence have reported that d-gal treatment induces activation of Iba-1 and GFAP in a mouse model of d-gal-induced aging [5, 50], in which neuroinflammation is triggered by the release of various proinflammatory mediators (TNF-α and IL-1βeta) from glial cells [1, 61]. Similarly, we previously reported that d-gal leads to p-JNK overexpression in association with activation and accumulation of numerous inflammatory mediators [1]. Here, we found a marked increase in the levels of TNF-α and IL-1βeta in the hippocampal region in the brains of d-Gal-treated mice compared to those of saline-treated mice, which was significantly reversed by d-gal + Gly cotreatment. In addition, we found that compared to d-gal alone, cotreatment with Gly and d-Gal markedly reduced the protein expression and immunofluorescence reactivity of GFAP and Iba-1 in the cortex and hippocampus. These findings indicate that Gly treatment is effective against d-gal-induced neuroinflammation via inhibition of JNK-mediated activation of glial cells and neuroinflammatory mediators.

Early studies linked oxidative stress and abnormal activation of the JNK signaling pathway with age-associated learning and memory deficits [62, 63]. Another study reported that brain functions largely depend on regulation of synaptic plasticity by synaptic proteins [39]. Importantly, d-gal treatment induced synaptic and memory impairment by downregulating memory-associated pre- and postsynaptic protein levels in the aging mouse brain [39, 64, 65]. Correspondingly, our results showed that d-gal treatment reduced the expression levels of PSD95, SYP and Syn, whereas cotreatment with Gly reversed this effect by significantly alleviating the d-gal-induced decreased in these memory-associated synaptic markers in the mouse hippocampus. Similarly, in the MWM test, we found that Gly treatment reduced the escape latency and increased the number of platform crossings and the amount of time spent by the mice in the target quadrant. Additionally, we found an increase in the percentage (%) of spontaneous alterations in the Y-maze task. Taken together, these findings indicate that Gly cotreatment improves behavior and memory by reducing d-gal-induced synaptic protein loss and spatial learning/cognitive impairment. Based on these findings, we suggest that Gly treatment is effective against d-gal-induced cognitive impairment by reversing JNK-mediated synaptic dysfunction and memory impairment.

## Conclusion

**Fig. 8:**
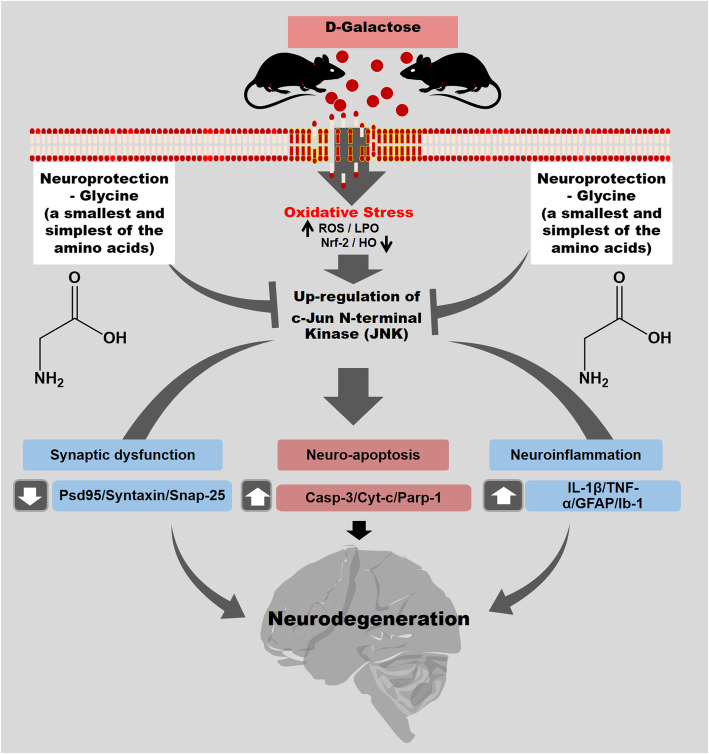
Suggested graphical representation of possible glycine neuroprotective mechanism against d-gal-induced neurotoxicity. Glycine (amino acid) via inhibition of c-Jun N-Terminal kinase reduced d-gal-induced oxidative stress (ROS), neuroapoptosis, neuroinflammation, synaptic dysfunction, memory impairment, and neurodegeneration induced by d-galactose in C57BL/6N mice

In conclusion, we demonstrate that treatment with Gly (the smallest and simplest amino acid) efficiently abrogates d-gal-induced oxidative stress-mediated c-Jun N-terminal kinase (JNK) activation, neuroapoptosis, neuroinflammation, synaptic dysfunction, and memory impairment in the brains of d-gal-treated mice (Fig. 8). Based on both molecular docking analysis and an in vitro study, we hypothesize that Gly, in addition to other well-known effects via NMDR receptor , our approach provides an additional/alternative neuroprotective mechanism of Gly against d-gal-induced neurotoxicity, possibly through inhibition of JNK protein function (binding ability + stable Gly-JNK protein interactions). Future detailed studies assessing the therapeutic efficacy and precise mechanisms of Gly are encouraged to provide evidence for this amino acid as an innovative therapeutic candidate for various neurodegenerative disorders.

## Data Availability

The authors hereby declares that the generated datasets in this study will be presented upon request from the corresponding author.

## Abbreviations

CNS: Central nervous system
JNK: c-Jun N-terminal kinase
MAPKs: Mitogen-activated protein kinase
TBST: Tris-buffered saline with Tween
PD: Parkinson’s disease
AD: Alzheimer’s disease
TNF: Tissue necrosis factor
PBS: Phosphate buffer saline
SDS-PAGE: Sodium dodecyl sulfate polyacrylamide gel electrophoresis
DAPI: 4′,6-Diamidino-2-phenylindole
TRITC: Tetramethylrhodamine isothiocyanate
FITC: Fluorescein isothiocyanate
Iba-1: Ionized calcium binding adapter molecule 1
GFAP: Glial fibrillary acidic protein
